# Epilepsy: The Quintessential Pathology of Consciousness

**DOI:** 10.3233/BEN-2011-0311

**Published:** 2011-03-29

**Authors:** Andrea Eugenio Cavanna, Fizzah Ali

**Affiliations:** ^1^Department of NeuropsychiatryUniversity of Birmingham and BSMHFTBirminghamUK; ^2^Sobell Department of Motor Neuroscience and Movement DisordersInstitute of NeurologyUCLLondonUK

**Keywords:** Epilepsy, seizures, consciousness

## Abstract

Alterations in consciousness are central to epileptic manifestations, and involve changes in both the level of awareness and subjective content of consciousness. Generalised seizures are characterised by minimal responsiveness and subjective experience whereas simple and complex partial seizures demonstrate more selective disturbances. Despite variations in ictal origin, behaviour and electrophysiology, the individual seizure types share common neuroanatomical foundations generating impaired consciousness. This article provides a description of the phenomenology of ictal consciousness and reviews the underlying shared neural network, dubbed the 'consciousness system', which overlaps with the 'default mode' network. In addition, clinical and experimental models for the study of the brain correlates of ictal alterations of consciousness are discussed. It is argued that further investigation into both human and animal models will permit greater understanding of brain mechanisms and associated behavioural consequences, possibly leading to the development of new targeted treatments.

## 1. Consciousness: A neuroscientific conundrum

Historically examined, analysed and explored from a multitude of perspectives, the multi-faceted concept of consciousness is the modern day academic enigma. Previously attacked by the realms of philosophy, religion and psychology, recent decades have conversely witnessed renewed interest from a neuroscientific front. Advances in human neuropsychology and functional imaging have exposed correlations between neural processes and facets of conscious experience [60]. This, in part, has contributed to the scientific drive for appropriate placement of subjective experience alongside electro-chemical interactions, into the field of empirical scrutiny.

Despite a host of controversial definitions and dynamic components, consciousness is of indisputable clinical relevance; serious acute cerebral involvement is inextricably linked with the function of consciousness [49]. Progressive injury parallels deterioration in consciousness ranging from sleep disorders and neurodegenerative dementias, steadily culminating in vegetative states and profound coma [17]. Epilepsy, in particular, has been regarded as providing a key opportunity to illustrate the neural mechanics of altered conscious states. Current classification regards epilepsy as a spectrum disorder, characterised by underlying dynamic and reversible brain dysfunction that generates variable and selective impairment of consciousness, ranging from minor impairment of attention to total behavioural unresponsiveness. The principal dichotomy central to current International League Against Epilepsy classification is the distinction between 'partial' seizures (involving focal brain regions or confined to one hemisphere) and generalised seizures (involving diffuse regions throughout both hemispheres) [25]. Within this dichotomy resides the major definitional criterion: impairment of consciousness differentiates between 'simple' and 'complex' partial seizures [5]. The pathophysiology of the epilepsies presents an ideal avenue by which to scrutinise abnormal neural activity alongside

ISSN 0953-4180/11/$27.50 © 2011 – IOS Press and the authors. All rights reserved associated alterations in consciousness thereby exposing brain mechanisms and permitting correlation with implicated anatomical structures [5,14,44].

This article commences with interpretation of definitional issues surrounding the concept of consciousness. A review of a well recognised bi-dimensional model for the study of ictal conscious state follows. A synopsis of current insights into the neural foundations underlying seizure sub-types (generalised tonic-clonic, absence and complex partial seizures) and specific clinical phenomenology of ictal consciousness concludes this review.

### 1.1. Defining consciousness

Recent decades have delivered an overwhelming mass of contributions endeavouring to discover, elucidate or re-evaluate consciousness. Attempts at definition have varied considerably over time as offerings have concurrently ranged a multitude of academic domains. The quintessentially subjective experience of consciousness, therein, evades unequivocal definition. Indeed, the use of the term may be regarded as context-dependent [60]. It is pertinent to emphasise, however, the distinction between consciousness and self-consciousness; the latter conversely embodies notions such as a propensity to embarrassment, self-detection, self-recognition, self-knowledge and awareness of awareness [60].

Within clinical practice physicians regularly apply terminology such as ‘dwindling’, ‘lapsing’ and ‘clouding’ of consciousness, rendering consciousness akin to the waking state. In other words, consciousness may be regarded as the ability to perceive, interact and engage with the environment and with other individuals [23,42,60]. Specifically, from the clinical perspective of an epileptologist consciousness is considered as the patient’s responsiveness during the ictal state, or conversely as ‘loss of contact’ with the surrounding environment. Yet this definition is far from all-inclusive, initially failing to account for alternative origins of sub-optimal responsiveness; whether ictal disturbance of speech, attention or other transient disturbances of memory and sensory processes [44,59]. This limitation is particularly crucial when considering seizure type. Generalised seizures are characterised by complete loss of consciousness, whereas complex partial seizures often cause selective and limited disturbances that may culminate into motor/sensory aphasia or transient inattention [27,41]; end-points that are open to misinterpretation as loss of consciousness. This simplistic definition further fails to consider variations in the subjective content component of consciousness. Therein, lies the concept of a bi-dimensional consciousness.

### 1.2. Level and contents of consciousness

Conventionally, neurology has widely employed the term consciousness to mean alertness or responsiveness [36], conversely theoretical approaches have regarded subjective experience central to the concept of consciousness [46]. An amalgamation of neurophysiological and neuroimaging studies indicate the division of the neural correlates of consciousness into structures necessary for the maintenance of the quantitative (level) of consciousness and those responsible for generating the qualitative (content) features of conscious experience [5,16,17]. Epileptic seizures involve changes to both the objective level of awareness and subjective content of consciousness.

The first element, the level of consciousness, may be considered loosely akin to the conventional meaning of consciousness i.e. arousal or vigilance. Specifically, it is a matter of ‘degree’ along a spectrum extending through a range of consciousness and unconscious states, from alertness continuing through into sleep and terminating in coma [38,58]. The level of consciousness may be further compartmentalised into multiple synergistic systems necessary for the maintenance of (1) alert state (2) attention (3) awareness of self and environment. The role of the ascending activating pontomesodiencephalic reticular formation in association with its thalamic projections is fundamental to the neurobiological basis of vigilance [45]. Functional imaging reports have further demonstrated subcortical spread of ictal discharge to consume the normal activity of thalamocortical networks, in turn generating complete loss of consciousness [40].

The second element, the content of consciousness, includes an array of multimodal perceptions (emotions, sensations, memories and intentions) and in itself may be regarded as a mutli-faceted dimension encircling sensorimotor, emotional and memory systems [5]. Subjective experiences are manufactured through interaction between exogenous environmental input and endogenous factors such as internal attention, and are governed by temporo-limbic activity [21,22,37]. Accordingly, the depth and the emotional salience of such experience demonstrates great variability, ranging from fringe phenomena to intense experiences. Previous investigations have noted the significance of the temporal cortex in inducing changes in the contents of consciousness [35], with further observations about the role of medial temporal lobe structures in the conscious recall of past events [29].

Consciousness is not a unitary phenomenon and having provided discourse on its individual dimensions, it is pertinent to comment on the extent to which the components are separable. The content of consciousness may be deemed the substrate system upon which level of consciousness acts [10]. The level of arousal holds considerable control over the contents of consciousness; in most instances an increase in arousal is accompanied by more vibrant conscious experience. Exceptional states such as limbic status epilepticus must be acknowledged, whereby high levels of arousal are coupled with depleted contents of consciousness. The union between level and content of consciousness is incompletely determined; disruption of either, however, engenders loss of consciousness. Cortical dysfunction is a mutual player in the disruption of consciousness: involvement of higher order association cortex yields impairment in the level of consciousness, and specific regions of the sensory or motor cortex induce alterations in the content of consciousness [10]. In the absence of generalised cerebral dysfunction, the contents of consciousness indicate the specialised function of particular brain structures.

Traditionally, evaluation of the ictal conscious state has involved observer interpretation and individual vocabulary, riddled with poor inter-rater and test-retest reliability and aggravated by an absence of standardised tools. Over recent years an integrated bi-dimensional model of consciousness has been proposed, reconciling qualitative and quantitative aspects of consciousness and allowing the comprehensive assessment of the ictal conscious state in accordance with these two key component elements.

### *1.3. The bi-dimensional model of consciousness*

Comprehensive assessment of ictal clinical alterations requires combined assessment of both the level and contents of consciousness. Graphical representation of the level of consciousness plotted against the content of consciousness allows delineation of both pathological and physiological conscious states [14,44] and provides a valuable framework for the comprehensive and systematic assessment of seizure-specific ictal conscious state. The waking state in a healthy subject is characterised by an almost invariably high level of arousal but less predictable contents of consciousness. Accordingly, generalised seizures are characterised by total absence of both responsiveness and subjective experience. Complex partial seizures are characterised by more selective disturbances to sensory processes, attention or memory and demonstrate variable impact on both the level and content of consciousness [17]. Seizures of temporal lobe origin display heightened subjective experiences as a result of experiential phenomena, typically combining elements of perception, memory and affect [19,28]. Simple partial seizures are accompanied by visual, auditory and somatosensory features that selectively impact on the contents of consciousness, preserving levels of awareness [17].

Within this paradigm a twenty-item self-report scale, the Ictal Consciousness Inventory (ICI) has been developed permitting quantitative analysis of the ictal state as per the established bi-dimensional representation. Patients with epilepsy complete one questionnaire per witnessed seizure recalled. The first ten items of the scale assess the level of general awareness/responsiveness; including general awareness of time, place and others; comprehension and responsiveness; gaze control; forced attention and voluntary initiative. The second set of items assess vividness of ictal subjective experience (content of consciousness), including dreamy states and derealisation, illusions and hallucinations, and ictal emotions. Each item is rated on a 0–2 Likert type scale and the scale yields two sub-scores for each dimension of consciousness each ranging from 0 to 20. The ICI reveals the nature of impairment in consciousness, allowing differentiation between those seizure types principally generating a deficit in the level of awareness (generalised seizures, including absence (*petit mal*) or tonic-clonic (*grand mal*)) and those modifying the contents of consciousness (focal seizures). Despite the intrinsic limitations of user compliance and ictal amnesia generating recall bias, the ICI features as a user-friendly tool permitting description of the ictal state in a systematic and standardised fashion [18].

Regardless of evident variations in ictal origin, behaviour and electrophysiology, the underlying neural foundations of impaired consciousness are bound by common anatomical structures.

## 2. A common foundation

Core to the pathology of epilepsy is the anomalous increase or decrease in neural activity that provokes abnormal brain function. Regional increases or decreases in neuronal activity tend to vary between absence, generalised tonic-clonic and complex partial seizures [5].

Though physiologically distinct, the seizure types are anatomically bound. Investigation into both normal states of arousal and circumstances of impaired consciousness (anaesthesia, sleep and brain lesions) has exposed a common network of cortical and sub-cortical structures involved in the generation of consciousness. This 'consciousness system' comprises regions of the upper brainstem (midbrain and upper pons), medial thalamus, cingulate gyrus, medial prefrontal cortex, precuneus, as well as lateral frontal and parietal association cortex. Additionally the significance of midline and intralaminar thalamic nuclei and hypothalamus as well as activating systems located in the basal forebrain have been noted [5]. The basal ganglia and cerebellum, implicated in the control of attention, may be cautiously included.

The following sections are designed to provide the reader with insight into the neuroanatomical architecture generating seizure specific alterations in consciousness.

### *2.1. Consciousness in generalised seizures*

#### *2.1.1. Generalised tonic clonic seizures*

Generalised tonic-clonic seizures (GTCS) or grand mal seizures are characterised by abnormal bi-hemispheric neuronal discharge generating complete unresponsiveness and convulsions. The sudden tonic phase of these seizures comprises sustained axial muscle contraction, upward eye deviation and pupillary dilatation, in association with 10–20 seconds of high frequency electroencephalographic (EEG) activity. The ensuing clonic portion refers to rhythmic limb contraction with EEG demonstrating poly-spike-and-wave discharges [5]. GTCS can be primarily generalised with no clear focal onset. Alternatively, discharges originating from a single focus may propagate to form secondarily generalised seizures (i.e. secondary generalisation of partial seizures). Nonetheless, both primary and secondary generalised seizures are typically accompanied by a 'black-out' of both awareness and subjective experience of consciousness.

Investigations into the neural correlates of impaired consciousness in GTCS signal the involvement of specific brain regions more intensely rejecting the idea of homogenous participation of the whole brain. Although initial neuroimaging studies indicated homogenous involvement of the entire brain [26] and animal studies based on electrophysiological, blood flow and metabolic mapping of GTCS have produced variable findings, other investigations have demonstrated more regional changes [5,17]. Single photon emission CT (SPECT) studies of cerebral blood flow in secondary GTCS have demonstrated focal involvement of the brain, frequently in the region of seizure onset [6,11,39,54]. Additionally, SPECT inter-ictal difference imaging of secondary GTCS as part of epilepsy and GTCS provoked by electro-convulsive therapy in patients with treatment refractory-depression has revealed cerebral blood flow increases during the seizure in particular brain regions. Those brain regions harnessed by the 'consciousness system' are implicated. Increased blood flow was seen in the thalamus and upper brainstem as well as lateral frontal and parietal cortex. Conversely, decreases in cerebral blood flow were seen during the seizure period in the cingulate gyrus. Post-ictal unconsciousness was accompanied, also, by diminished blood flow in the anterior and posterior cingulate gyrus as well as the lateral frontal and parietal cortex. Inhibitory cerebellar outputs are potential players in seizure termination and in diminished consciousness post-ictally; increases in cerebral blood flow are coupled with increased flow in the thalamus and with substantial decrease in blood flow in frontal and parietal cortex [5,17,48].

The bilateral frontal and parietal association cortices are associated with greatest increases in cerebral blood flow during GTCS. The focal disruption in GTCS of these loci together with effects on related sub-cortical structures yields the intense and persevering impaired consciousness typical of GTCS. Conversely, there is relative sparing of the intervening regions of the primary sensory and motor cortices. Additionally, rare seizures limited to the bilateral sensorimotor areas [47] and secondary GTCS following spread from a temporal lobe focus [4] exhibit total preservation of consciousness.

#### *2.1.2. Absence seizures*

Absence seizures are typified by sudden onset of blank staring and unresponsiveness, usually lasting 5–10 seconds. Interruption or cessation of voluntary behaviour, as well as a variety of mild motor manifestations ranging clonic, atonic, tonic, or myotonic activity may also feature. Typically eyelid fluttering or myoclonic jerks are witnessed, yet, the motor manifestations of absence seizures are subtle; allowing absence seizures to pose as the most 'transparent' form of ictal impaired consciousness. Recovery is sudden with previous activity resuming and no significant post-ictal deficit [17]. Absences are generated through abnormal cortico-thalamic network oscillations whereby the thalamus acts as the generator of the 3Hz oscillation and the cortex generator of the spike and wave patterns. In combination the characteristic bilateral and frontal pre-dominant 3–4 Hz large amplitude spike-and-wave discharges result [56]. Although absences are regarded as essentially 'generalised', both human and animal studies indicate the specific involvement of particular cortico-thalamic networks with relative sparing of other regions. Earlier human imaging studies, marred by technical limitation of method and the intrinsic variability of absence seizures themselves [10], yielded incongruent findings. Some investigations demonstrated global increases in cerebral blood flow, whilst others have shown no change or alternatively increased or decreased blood flow in either focal or generalised patterns [5,17].

Preliminary investigation through functional MRI (fMRI) with simultaneous EEG recordings (EEG-fMRI) has verified that generalised spike-wave seizures selectively involve particular networks, whilst sparing others. The improved spatial and temporal resolution has further allowed identification of bilateral thalamic activation, and cortical signal decrease in the anterior and posterior midline regions, and lateral frontal and parietal association areas [1,30,33,53]. A number of these studies also noted signal increases in the lateral frontal and parietal cortical regions. Accordingly, various authors have discounted the impaired consciousness of absence seizures as a whole brain phenomenon. Alternatively it has been postulated that disruption of normal information processing in specific brain regions, namely the bilateral fronto-parietal association cortex as well as related sub-cortical structures is implicated [5,17].

### 2.2. *Consciousness in complex partial seizures*

Partial epileptic seizures are of focal origin; abnormal discharges may remain localised to the original source or can secondarily generalise. Partial epileptic seizures can be further classified into simple partial seizures whereby consciousness is retained, or alternatively complex partial seizures which are defined by a loss of consciousness. Clinical manifestation is dependent on cortical origin, extent of seizure propagation and duration.

Complex partial seizures usually last between 15 seconds and 3 minutes and demonstrate variable alterations in the consciousness state. Characteristically, consciousness is initially spared, seizure progression however brings variable loss of contact with the environment with most profound impairment typically late in the seizure, persisting for several minutes post-ictally. The temporal lobe is the original seizure focus in approximately 80% of patients with partial seizures [13] and mesial temporal lobe sclerosis features as a frequent neuropathological association [57]. Focal premonitory phenomena such as fear, rising abdominal sensation or lip smacking automatisms (epileptic aura) mark seizure onset [2]. As the seizure evolves, clinical manifestations ranging affective, mnemonic and composite perceptual phenomena are experienced. Affective components of experiential phenomena ('epileptic qualia') [44], include pleasant (joy, excitement, euphoria) and unpleasant (sadness, fear, guilt) subjective feelings, symptoms of depersonalisation (altered sense of self) and derealisation (altered experience of the external world). Cognitive and psychosensory subjective experiences may feature [3,34] as well as auditory distortions, metamorphopsias, olfactory and gustatory hallucinations. Automatisms (stereotyped behavioural patterns) including oral activities such as lip smacking, chewing, swallowing, as well as postural changes which can accompany or follow experiential phenomena. The limbic components of the medial temporal lobe, in particular the amygdala, have been related to the affective component of experiential phenomena [17, 44]. However, the vast array of experiential phenomena indicates recruitment of wider neural networks beyond the temporal lobe.

SPECT and EEG studies indicate the requirement of bilateral temporal lobe involvement for loss of consciousness (general level of awareness) in complex partial seizures [3]. Although bilateral temporal lobe dysfunction generates amnesia, we resort to the 'network inhibition hypothesis' to fully explain the association between impaired consciousness and focal temporal lobe seizures [48]. In accordance with this model, medial temporal lobe seizures propagate to midline sub-cortical structures (medial diencephalon and ponto-mesencephalic reticular formation), interrupting their normal activating function, in turn spawning widespread inhibition of non-seizing regions of the frontal and parietal association cortex. Fronto-parietal network inhibition may be ultimately accountable for the impaired level of consciousness in the late ictal and immediate post-ictal phase. It is pertinent to note that the structures listed in this hypothesis are those comprising the consciousness system. SPECT has further shown complex partial seizures with impairment of consciousness to exhibit increases in cerebral blood flow in the upper brain stem and medial thalamus, and hypoperfusion in the fronto-parietal association cortex and the anterior and posterior inter-hemispheric regions. This model was scrutinised through ictal-interictal investigation of 24 subjects with surgically confirmed mesial temporal lobe epilepsy through analysis of cerebral blood flow changes whilst performing continuous video-EEG monitoring [8]. Complex partial seizures (associated with loss of consciousness) displayed widespread changes; increases in cerebral blood flow in the temporal lobe and in midbrain subcortical structures (including mediodorsal thalamus and upper brainstem), alongside decreases in the frontal and parietal association cortex bilaterally (including lateral prefrontal, anterior cingulate, orbitofrontal and lateral parietal cortex). Simple partial seizures (characterised by sparing of consciousness) in contrast, were accompanied by more focal changes limited to the temporal lobes. Furthermore, EEG recordings obtained during temporal lobe seizures with impaired responsiveness show marked slowing in bilateral frontal and parietal association cortices, noted to be particularly prominent in the late ictal phase and extending into the early postictal period [9]. Thus supporting the hypothesis that abnormal activity in the midline cortical structures (upper brainstem–diencephalic activating systems), which disseminates into diffuse inhibition of the fronto-parietal cortex, causes loss of consciousness during temporal lobe seizures.

### *2.3. Epilepsy and the brain ‘default mode’*

A higher order associative network consisting of the lateral parietal, ventromedial prefrontal, mid-dorsolateral prefrontal and anterior temporal cortices along with the postero-medial parietal region is highly metabolically active during the resting state (lying quietly awake with eyes closed). The posteromedial cortex comprises retrosplenial cortices, the posterior cingulate gyrus as well as the precuneus [51]. The precuneus acts as an integral component in four general functions: body movements in space, episodic memory retrieval, visuo-spatial imagery, self-awareness and, pertinent in this instance, consciousness. It is of particular interest as it is the most active of the three regions, consuming approximately 35% more glucose [32]. The tonic level of activity of this neural network decreases with engagement in goal-directed cognitive or perceptual tasks, otherwise termed task-induced deactivations (TIDS). These findings may be translated into the ‘default mode’ of brain function, whereby the demand of focused attention on a particular task heralds curtailing of baseline brain processes such that deactivations in the regions serving the broad information gathering of the baseline state are observed [32,43]. Functional imaging studies have further demonstrated significant deactivation of ‘default mode’ network of activity in altered states of consciousness including slow wave as well as rapid-eye movement sleep, general anaesthetic induced sedation and the persistent vegetative state [15,19]. Likewise, several neuropsychiatric conditions including Alzheimer’s disease, schizophrenia and attention-deficit hyperactivity disorder have also been linked to deactivations in ‘default mode’ zones [15,31]. Wholly, these findings suggest the existence of this multimodal associative network, to which the precuneus is integral, to function as part of greater neural machinery sub-serving self-awareness and conscious experience i.e. baseline backdrop processes running during a quiet resting state [15,19,52,55].

## 3. Conclusion

Seizure activity has been long associated with alterations in consciousness, indeed, transient impairment in consciousness has been heralded as indicative of generalised seizure activity [27,59]. Epileptic seizures form a spectrum of disorders that generate selective, variable and transient impairment of consciousness, ranging from minor impairment of attention to total behavioural unresponsiveness. The immense variability in ictal semiology offers an ideal opportunity by which to examine the associated underlying pathological neurological function. Through an established bi-dimensional model, evidenced by neurophysiological and neuroimaging studies, this article has sought initially to delineate this spectrum of seizure-specific consciousness alterations and secondly to clarify the underlying neural correlates. The level of awareness and contents of consciousness are variably affected by different epileptic seizure types.

A collection of neuro-imaging studies demonstrate that generalised seizures do not encompass all brain areas homogenously. Likewise, complex partial seizures have been noted to be less focally active than previously recognised [7,8,10,12]. Moreover, an accumulation of evidence indicates a common network, the ‘consciousness system’, overlapping with the ‘default mode’ network, between all seizure sub-types. The upper brainstem and medial thalamus, the anterior and posterior cingulate, lateral and orbital frontal cortex and lateral parietal cortex as well as the medial frontal cortex and precuneus are mutually implicated. Investigation into specific neurobiological correlates allows further development of targeted treatment, including new pharmacological therapy as well as deep brain stimulators. It is imperative that such new treatments aim to preserve consciousness as it is within this impairment that the negative outcomes of seizures (accidents, trauma, driving and occupational sanctions) flourish.
